# Versatile direct laser writing of non-photosensitive materials using multi-photon reduction-based assembly of nanoparticles

**DOI:** 10.1038/s41598-019-50630-1

**Published:** 2019-10-04

**Authors:** Hiroaki Nishiyama, Kan Umetsu, Kaito Kimura

**Affiliations:** Graduate School of Science and Engineering, Yamagata University, 4-3-16, Jonan, Yonezawa, Yamagata Japan

**Keywords:** Laser material processing, Nanoscience and technology

## Abstract

Versatile direct laser writing (DLW), not limited by material photosensitivity, offers opportunities for fundamental and technological innovation for micro-/nanofabrication in integrated photonics, electronics and material science. Although DLW has high potential in micro-/nanodevice fabrication, material choice suffers an intrinsic limitation: DLW cannot be applied to non-photosensitive materials. We describe a newly discovered rapid-assembly phenomenon of fine particles based on femtosecond laser multi-photon-reduction in solution. This phenomenon allowed the writing of micropatterns with thick clad layers filled with nanoparticles. We wrote continuous patterns by moving the laser focus even in the case of non-photosensitive material such as SiO_2_. By transcending the strict material limitation, this novel laser writing process promises to be a powerful tool in a variety of scientific fields.

## Introduction

Versatile direct laser writing (DLW), not limited by material photosensitivity, offers the possibility of true photo-fabrication of highly functional micro-/nanodevices, which is of interest in various fields such as nanophotonics, electronics, material science and life science. DLW is a simple and rapid fabrication process for various micro-/nanostructures based on light-matter interaction including photo-polymerisation, photo-reduction, sintering, lattice defect formation, phase separation and carbonization^1–6^. Micro-/nanostructures can be written only by translating a laser focus. Compared to DLW, conventional lithographic fabrication requires complicated and repetitive processes such as vacuum deposition and plasma etching. So far, many groups have reported DLW of Ag, Au, Cu, photopolymers, carbon, photosensitive glasses, modified polydimethylsiloxane and so on^7–15^. Although it has succeeded in the formation of functional structures for nanophotonic devices^16–20^, super capacitors^21^, microactuators^22^, superhydrophobic surfaces^23^, and bio-scaffolds^24,25^, the technique typically encounters an intrinsic material limitation. That is, DLW cannot be applied to materials without photosensitivity. Adequate optical responses to light sources are essentially required in target materials. In one use case, Ag micropatterns can be directly formed by laser irradiation of visible wavelength to Ag nano-ink^26^. In this process, laser light is absorbed by Ag nanoparticles, followed by photothermal reactions to form the patterns. However, it is not easy to apply this laser set-up to DLW of other materials such as different metals because these other targets do not necessarily have appropriate absorption properties and chemical reaction paths at this laser wavelength. That is, laser writing capabilities and properties including pattern size and height strongly depend on incident laser wavelength. From such a material limitation, it is also difficult to obtain SiO_2_ micropatterns because of its high transmittance over a wide wavelength range. Therefore, vacuum UV photon such as F_2_ laser (157 nm wavelength), which has photon energy higher than SiO_2_ bandgap, is often required for glass processing^27,28^. In another example, to write signal filters, so-called fiber Bragg gratings, inside optical fiber cores, KrF excimer laser of 248 nm wavelength is used because this wavelength fortunately matches an intrinsic absorption band of the fiber core materials^29,30^. Such optimal irradiation ultimately allows for structural changes in Ge-related defects, resulting in refractive index changes and density modification at the irradiated region^4,30^. Conversely, excessive absorption causes serious thermal damage just beneath the material surface because of the short penetration depth of light. Thus, an adequate overlap between laser oscillation wavelength and absorption band is a key factor for DLW. Of course, light–matter interactions can also be started by nonlinear optical absorption induced by ultrashort pulse laser^31–33^. However, even for such cases, sufficient nonlinear responsivity and absorption intensity of materials is required. Although DLW has high potential for a variety of applications, a strategy to overcome the strict limitation on material choice remains elusive.

In this study, we report the discovery of an effective assembly phenomenon of nanoparticles in solution based on femtosecond laser multi-photon-reduction. The most important feature of this process is that much wider range of material choices in DLW was achieved, which allows us to form micropatterns regardless of the material photosensitivity. The laser-written structure exhibited a hierarchical cross-section with unique clad layers filled with the fine particles. Continuous micropatterns were obtained by moving the laser focus at low power. Herein, we report the assembly properties of several kinds of nanoparticles and discuss the mechanism of this phenomenon.

## Results

### Formation of hierarchical structures

**Figure 1:**
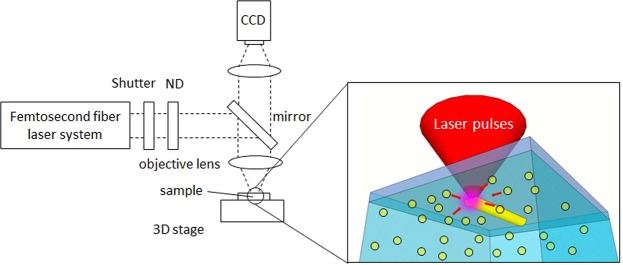
Laser irradiation set-up for DLW.

Three types of nanoparticles were dispersed to a dilute AgNO_3_ solution with each solution containing one nanoparticle type: TiO_2_, SiO_2_ and Fe_2_O_3_. Average diameters of TiO_2_, SiO_2_ and Fe_2_O_3_ nanoparticles were, respectively, 20 nm, 22 nm and 110 nm. Figure 1 is a schematic illustration of the laser irradiation set-up for DLW. The solution was inserted between a Teflon chamber and a substrate. Femtosecond laser pulses were focused on substrate surfaces. The pulse duration, center wavelength and repetition rate were, respectively, 127 fs, 780 nm and 100 MHz. Micropatterns were written by moving the laser focus using a computer-controlled three-axis stage system.

**Figure 2:**
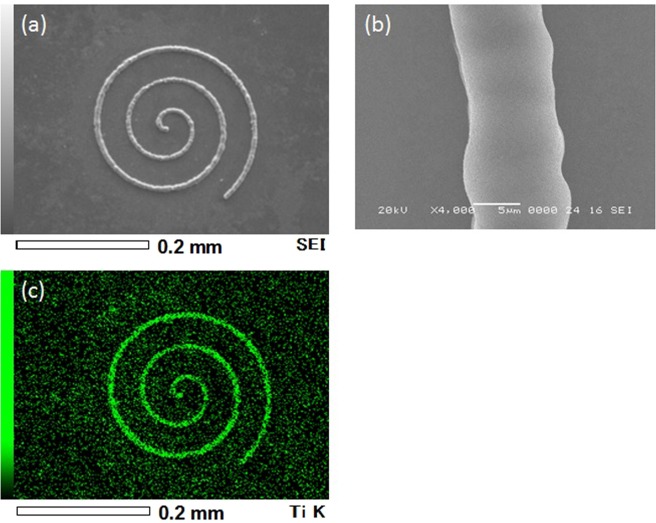
(**a,b**) SEM images and (**c**) elemental map of a micro spiral pattern fabricated by DLW in the solution with TiO_2_ nanoparticles.

**Figure 3:**
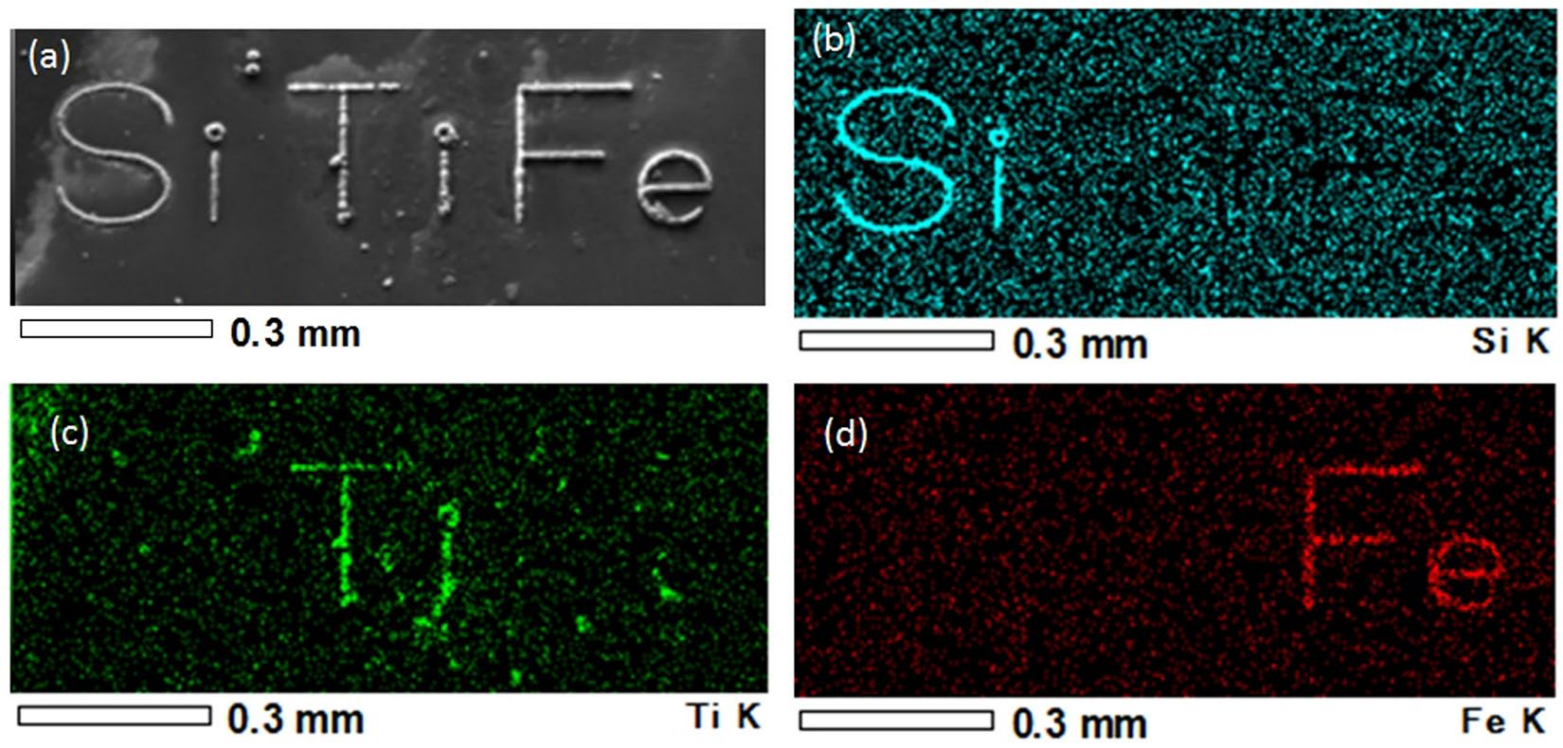
(**a**) SEM images and elemental maps of micro-characters of (**b**) “Si”, (**c**) “Ti” and (**d**) “Fe” on a CaF_2_ substrate.

Figure 2 shows SEM images (a and b) and elemental map (c) of a micro spiral pattern on a substrate formed by translation of laser focus in the solution with TiO_2_ nanoparticles. Here, laser power and laser writing speed were 30 mW and 40 μm/s, respectively. The laser writing process was observed from the upper side by CMOS camera system in real time. A movie of typical laser writing is provided in the supplementary information. Each sample was washed by ethanol after laser writing and dried by N_2_ gas stream. A continuous micropattern with smooth surfaces was formed by moving the laser focus. There were almost no cracks on surfaces at this writing condition. From scanning electron microscopy with energy dispersive X-ray spectroscopy (SEM-EDX) analysis, intense signals of Ti were detected from the lines. Maximum laser writing speeds as high as 900 μm/s were achieved. In addition to the solution with TiO_2_ nanoparticles, we also obtained micropatterns using other solutions containing different nanoparticles. Figure 3(a) is micro-characters of “Si”, “Ti” and “Fe” on a CaF_2_ substrate. These characters were, respectively, written using the abovementioned three nanoparticle solution types: SiO_2_, TiO_2_ and Fe_2_O_3_ at 30 mW and 50 μm/s. The EDX analysis illustrated in Fig. 3(b) indicated that higher concentrations of Si, Ti and Fe were observed in the three micro-characters.

**Figure 4:**
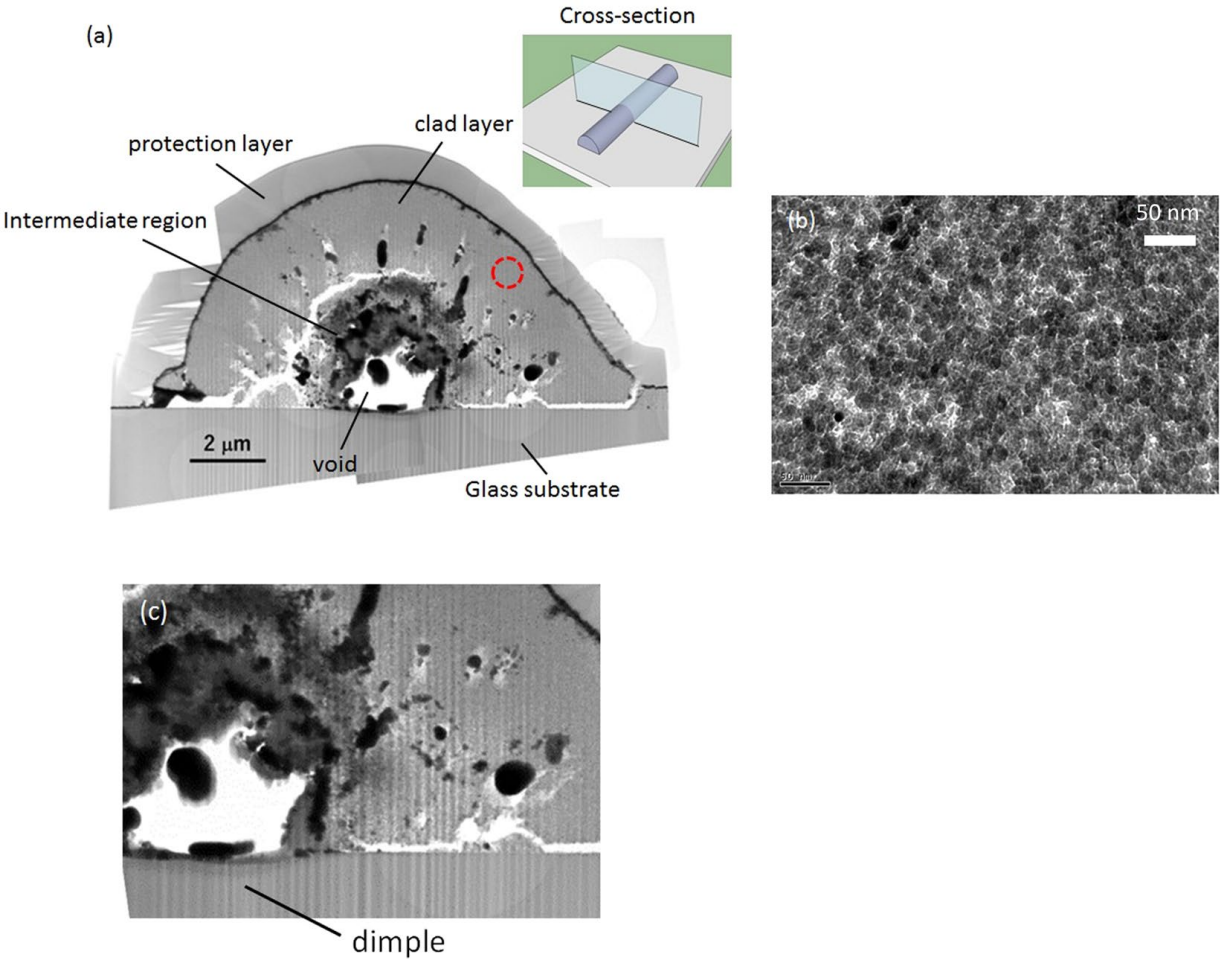
(**a**) Cross-sectional TEM image of line structures from the solution of TiO_2_ nanoparticles, (**b**) enlarged image of the clad layer filled with the nanoparticles (red dot circle in (**a,c**) enlarged image of the void-substrate interface.

Figure 4(a) presents a cross-sectional TEM image of line structures from the solution of TiO_2_ nanoparticles. The structure exhibited a hierarchical cross-section consisting of three regions: a void at centre, a thick clad layer and an intermediate region. The outermost layer is a protective film for FIB etching process. A slight dimple was observed at the substrate surface just beneath the void (Fig. 4(c)).

In particular, it should be noted in the enlarged image (Fig. 4(b)) that the clad layer consists of closely packed nanoparticles of diameter of approximately 20 nm. We observe a relatively clear borderline between the clad layer and the intermediate region. In this image, the micropattern had a semi-circle cross-section of 12 μm width and 6 μm height. The void diameter and the clad layer thickness were approximately 2 and 2.4−2.9 μm, respectively.

**Figure 5:**
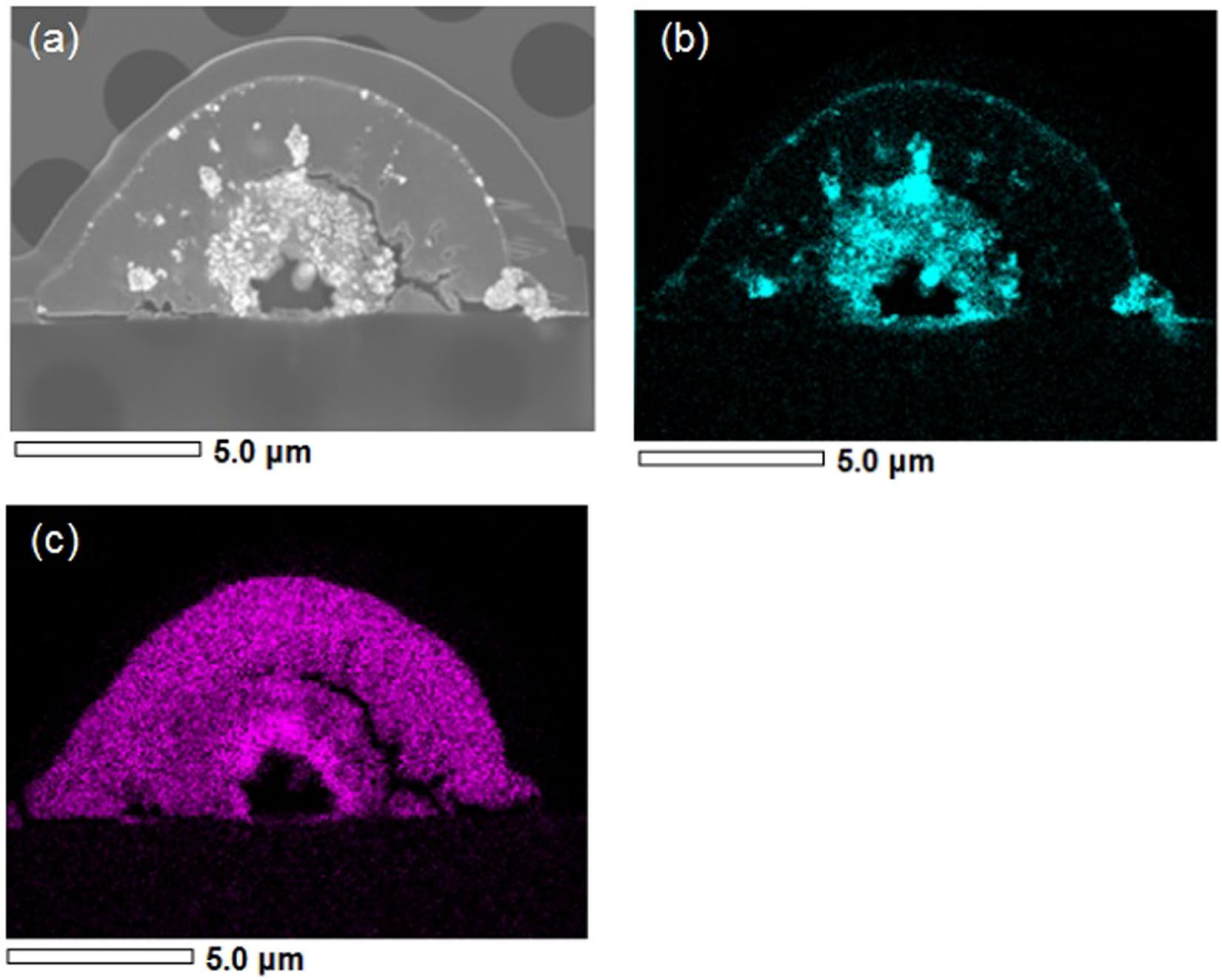
(**a**) Cross-sectional SEM image and elemental maps of (**b**) Ag and (**c**) Ti of a line structure from the solution of TiO_2_ nanoparticles.

Figure 4(a) depicts many dark spots of diameter 100−500 nm in the intermediate region. Chemical composition measurements shown in Fig. 5 revealed that dark spots in the intermediate region are composed of Ag, with composition separated along the borderline. In the intermediate region, Ag, Ti and O atoms were abundant. In comparison, the clad layer mainly consisted of Ti and O, with almost no Ag included. Furthermore, the stoichiometric proportion of Ti and O was measured to be approximately 1:2, meaning that the clad layer was an aggregated structure of TiO_2_ nanoparticles. Such unique hierarchical structures were obtained not only for the solution of TiO_2_ nanoparticles but also for those of SiO_2_ and Fe_2_O_3_ nanoparticles. The nanoparticles in each clad layer are responsible for the elemental signals detected in Fig. 3(b–d). The micropatterns adhered to substrates so rigidly that they were not peeled away even after cleavage of the substrates. Such stability seems originate from the mechanical support by a Ag-based core.

This laser writing process has two significant advantages over previous laser methods. First, this phenomenon does not necessarily depend on optical properties of the nanoparticles. The optical band gap of SiO_2_ is more than 7.8 eV, which is nearly 5 times higher than that of the incident photon energy of 1.59 eV. By contrast, Fe_2_O_3_ has intense absorption over the visible range up to 560 nm wavelength, which corresponds to a band gap of 2.2 eV. In spite of such large differences in optical properties, we obtained the clad layer of the nanoparticles under similar laser irradiation conditions at low intensities. Second, the clad layers were continuous, not isolated, along the laser translation direction. If the nanoparticles have conductive or dielectric properties, the clad layers can work as electric paths or electrical isolators, respectively.

### Laser writing property

**Figure 6:**
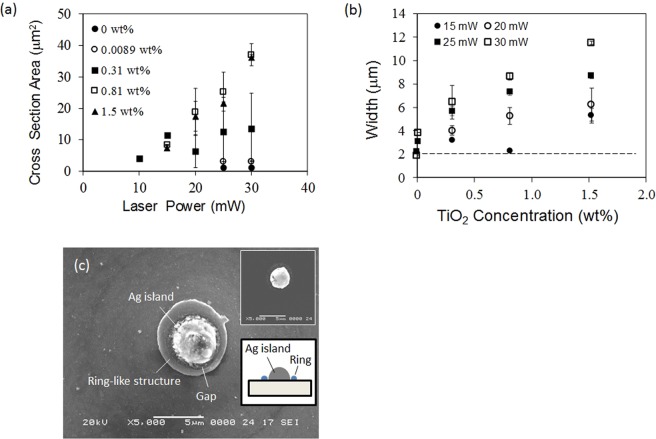
(**a**) Laser power dependence of the cross-section area as for the solution with TiO_2_ nanoparticles, (**b**) relationship between line width and TiO_2_ concentration as a function of laser power and (**c**) a SEM image of a ring-like microstructure around Ag island. Dot line in (**b**) is a diffraction limit. Inset of (**c**) is Ag island before irradiation.

Figure 6(a) is the dependence of the cross-sectional area on laser power for the TiO_2_ nanoparticle solution. The cross-sectional area refers to semi-circular region including the void, the clad and intermediate regions. The laser writing speed was kept at 30 μm/s for all runs. The cross-sectional area increased markedly with laser power. This tendency was more significant for the solution with a higher concentration of nanoparticles. At a laser power of 30 mW, the solution with 0.81 wt% TiO_2_ exhibited a cross-sectional area 11 times larger than that of the solution with 0.0089 wt% TiO_2_. Although the addition of nanoparticles enhanced the pattern formation, there was an effective threshold of laser power for DLW. We could not obtain continuous structures at power below this threshold.

Figure 6(b) illustrates the relationship between line width and TiO_2_ concentration as a function of laser power. The solution without nanoparticles exhibited the line widths less than 3.8 μm, which were similar to the diffraction limit of 1.9 μm. Compared to this, line width for solution with nanoparticles exhibited noticeable increases with increasing TiO_2_ concentration. For example, the solution with 1.5 wt% TiO_2_ produced a line width as large as 11.3 μm at the power of 30 mW, which corresponds to nearly 6 times the diffraction limit.

## Discussion

To understand the formation mechanism of the hierarchical structures, we focused attention on the borderlines between the core and clad regions. The core region denotes the region consisting of the intermediate region and the void. Such borderlines strongly suggest that the core and clad regions were formed based on distinct mechanisms. When femtosecond laser pulses are focused into AgNO_3_ solution without nanoparticles, multi-photon reduction of Ag ion is known to occur near the focal volume, resulting in Ag precipitation at the sizes similar to that of the laser spot^34^. In fact, similar results were obtained in our experiments on the solution without nanoparticles. In this case, line widths were obtained to be 2.0−3.8 μm (Fig. 6(b)). The slight discrepancy from the diffraction limit is caused by thermal reduction of Ag ion^35^. Considering that the core region of the hierarchical structure had a diameter similar to that of the laser spot and high Ag concentration, it is reasonable to suppose that the cores were generated by multi-photon reduction. Contrarily, the clad layer was positioned outside the laser focal spot. If such clad layers were formed based on thermal reduction of Ag ion or diffusion of radicals from the photo-reduction area, then the cross-section of the clad layers should have shown a continuous gradient of Ag concentration along the radial direction. However, since composition was clearly separated, diffusion processes must not be the predominant phenomena for clad layer formation. Therefore, increases of the cross-sectional area are likely to originate from deposition of nanoparticles onto the cores.

Average inter-particle distance was estimated to be 80 nm in the solution with 0.81 wt% TiO_2_. Considering that the nanoparticle diameter was 20 nm, the nanoparticle concentration was condensed simply by a factor of 64 (= 4^3^) in the clad layers, suggesting that there was a mechanism that rapidly attracted nanoparticles from a relatively large space around the laser spot.

**Figure 7:**
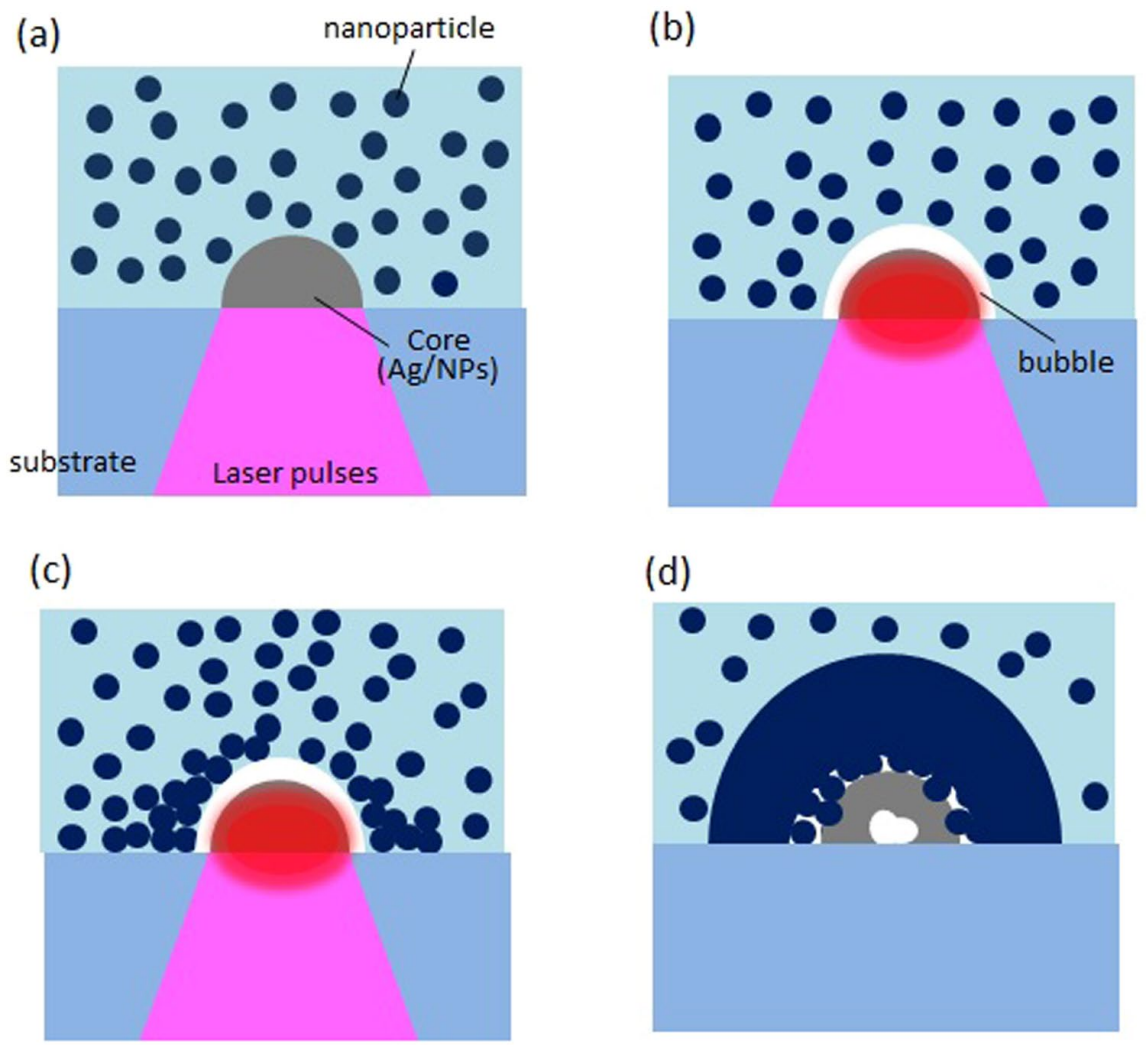
Potential mechanism of the assembly phenomenon of nanoparticles based on multi-photon reduction. (**a**) Ag-based core formation by multi-photon reduction, (**b**) bubble generation around the core by heat accumulation, (**c**) assembly of surrounding nanoparticles, (**d**) clad layer formation.

Figure 7 illustrates a potential mechanism for the formation of the hierarchical structures. First, Ag microdots are precipitated near the laser focus by multi-photon reduction of Ag ion. Here, nanoparticles inside the focus are buried into the Ag dot matrix, resulting in composite microstructures being the cores. Highly repetitive laser pulses of 100 MHz induce local heat accumulation in these composites, leading to microbubble generation around the composite. Several groups have reported the assembly of nanomaterials using laser-induced microbubbles in solutions^36,37^. In their studies, nanomaterials were drawn to gas/liquid interfaces of the microbubble by the Marangoni convection flow, which was induced by surface tension gradients along the bubble surfaces and by capillary convection. It is likely that similar flow-based effects in our assembly phenomenon were driven by microbubbles around the heated Ag-based core. In such a case, the core should have enhanced heat generation as an effective photothermal converter.

Based on the repetition rate of laser oscillator, the time interval between the laser pulses is estimated to be 10 ns, which is sufficiently shorter than the time required for heat to diffuse out of the focal spot (≈1 μs)^38^. Therefore, the core cannot return to room temperature before the next pulse arrives, leading to heat accumulation. Localised melting of oxide glass bulks with softening temperature exceeding 1000 °C was previously demonstrated by such photothermal conversion even at repetition rates less than 1 MHz^39^. The void and substrate deformation might be a trace of the local temperature increase of the core during irradiation. The transition temperature of the cover glass is approximately 550 °C. Therefore, it suggests that significant heating occurred locally at the interface of the Ag-based core and the substrate.

To investigate the assembly behavior of the nanoparticles around the cores, laser irradiation to Ag islands was carried out. First, Ag islands of 3 μm diameters were fabricated by laser focusing onto the substrates in AgNO_3_ solution of 0.4 M without nanoparticles, i.e. merely multi-photon reduction. Then, the Ag islands were placed in SiO_2_ nanoparticle-dispersed solution without Ag ions, followed by laser irradiation at 30 mW for 6 ms without moving the laser focus. Here, the concentration of SiO_2_ nanoparticles was 0.80 wt%. Figure 6(c) is a SEM image of the Ag island after irradiation. It should be noted that ring-like microstructures, with small gap from the Ag island, comprising a number of SiO_2_ nanoparticles appeared around the island. In previous reports on nanomaterial assembly using laser-induced microbubbles, ring-like microstructures were formed after disappearance of the microbubble^36,40,41^. Such structures were caused by the aggregation of nanomaterials, drawn to the bubble-substrate interface by convection flow. The ring-like structures strongly suggest that similar bubble-based assembly phenomenon occurred around the locally heated Ag-based cores, which is consistent with the proposed mechanism. In addition, Ag islands were fully covered by nanoparticles like domes in case of solutions with higher concentration of SiO_2_ nanoparticles. The irradiation time of 5 ms, which is the minimum switching time of our setup, corresponds to that per point in a laser scanning speed of 400 μm/s with spot diameter of 2 μm. The details of the assembly behaviors such as nanoparticle concentration dependence are currently under investigation, and will be reported in the future.

Dynamics of laser-induced vaper microbubbles around metallic heat sources have been reported by several groups^42,43^. These results show that the time for such bubble growth and shrinkage are approximately 10 ms, respectively. Convection develops with time delay ${l}^{2}/\nu$ where *l* is the characteristic size and *v* is the kinematic viscosity of the solution. Assuming that *l* is less than 100 μm, the time delay was estimated to be within 10 ms using *v* = 10^−6^ m^2^/s for H_2_O.

These results show that the assembly duration of nanoparticles, i.e., time difference between bubble lifetime and the time delay above, is considered to be relatively short (≈ several milliseconds). In contrast, flow speeds as high as 1 m/s were found to be generated in the vicinity of laser-induced microbubbles with a temperature difference of ∼300 K^42^. Such high-speed flow and unsteady convection flow might have significantly enhanced nanoparticle transport.

One candidate mechanism is a laser-induced microassembly without microbubbles. Laser microdeposition of polymer molecules was previously reported to be induced by optical trapping of molecular aggregates followed by liquid surface deformation^44^. In this case, the temperature increase of the aggregates was estimated to be as small as 2.4 K. Therefore, microbubbles were not generated by this laser irradiation. Another group suggested that enhanced mass transfer of molecules by Marangoni convection played a crucial role for this microdeposition^45^. In this phenomenon, although laser irradiation promotes the collection of the dissolved molecules into the laser focus by the electric field intensity gradients and convection flow, the optically induced surface deformation of the liquid conversely hinders further molecule assembly because of liquid removal from the trapped aggregates. Such conflicting effects lead to the laser power dependence of the deposition volume with a parabolic curve^44^.

The bubbles can remove the surrounding liquid from the laser focus, which is similar to liquid surface deformation mentioned above. Nevertheless, our assembly process exhibited an almost linear relationship between laser power and cross-sectional dimensions even at the writing condition where bubble generation was suggested (Fig. 6(a)). Such different behavior means that our assembly process is based on different mechanism, and that the microbubble around the core had a further role in addition to the removal of the solution. This result supports the proposed mechanism.

A second candidate mechanism, nonlinear optical absorption, has allowed for direct interaction between laser and non-photosensitive materials^31^. When near-infrared femtosecond laser pulses are focused into SiO_2_ glasses, photochemical structural changes occur around the laser spot. However, laser peak intensities of approximately 10^14^ W/cm2 were required in such nonlinear optical processes in solids^46^. By contrast, the peak intensity was 8.0 × 10^10^ W/cm2 in our experiments, which was several orders of magnitude smaller to induce nonlinear absorption of solid-state materials. Indeed, no changes were observed when laser pulses were focused to TiO_2_ and SiO_2_ glasses in our irradiation conditions even for several minutes. Therefore, the nonlinear interactions between laser pulses and nanoparticles appear to be negligible.

**Figure 8:**
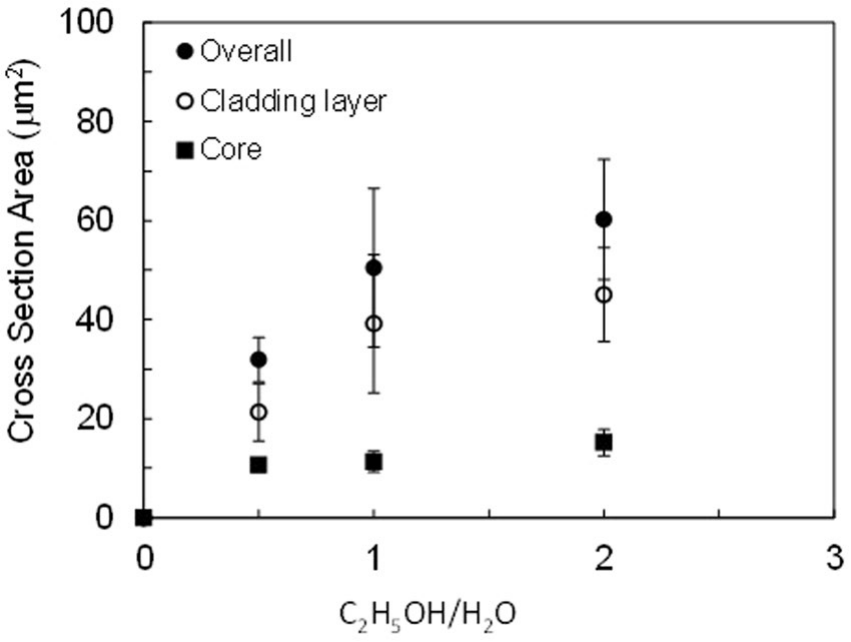
Changes in cross-sectional areas of the hierarchical structures as for TiO_2_ nanoparticle dispersed solution at different C_2_H_5_OH/H_2_O ratio.

In the proposed mechanism, laser-induced microbubbles play an important role in assembly. Figure 8 illustrates changes in cross-sectional areas of the hierarchical structures for different C_2_H_5_OH/H_2_O ratios in the solution. The example in Fig. 8 focuses on solution containing TiO_2_ nanoparticles, with the concentrations of AgNO_3_ and TiO_2_ kept constant in each solution. Latent heat of evaporation of C_2_H_5_OH (39 kJ/mol) was smaller than that of H_2_O (44 kJ/mol), meaning that an increase in C_2_H_5_OH ratio promotes generation of microbubbles. Whereas core cross-sectional area plateaued at 15 μm^2^ with respect to C_2_H_5_OH ratio, the whole area markedly increased to more than four times larger than the size of the core. This behavior means that addition of C_2_H_5_OH enhanced nanoparticle deposition on cores of similar size. Such rapid growth can be understood as effectively promoting larger bubbles, which have longer bubble life time, in a C_2_H_5_OH-richer solution. Conversely, no precipitation was observed in case of the solution without C_2_H_5_OH. This is because the reduction of Ag ion as a trigger of the assembly was not induced, indicating that Ag precipitation is essential for the cladding of nanoparticles. Although the volume of the Ag-based core could be controlled mainly by the laser spot size, whole volume of the hierarchical structure was not always determined only by such photochemical process. We should pay attention to the condition for the microbubble generation such as temperature increase of the core for optimization of DLW.

We also note that the void size tended to grow with increasing C_2_H_5_OH ratio. Microbubble generation results in isolation of the core from surrounding liquid. Higher concentration of C_2_H_5_OH apparently induced this isolation at an earlier stage of laser irradiation. Such heat insulation seems to have accelerated void formation, accompanied by temperature increase in the core. By means of this local temperature increase, the core seems to have been separated to two regions: the void and the intermediate region (Fig. 4(a)).

The laser-induced bubble process, which does not require direct interaction with nanoparticles, allowed us to create fine structures of various materials. By providing a much wider range of material choice, DLW can be a powerful tool for truly functional device fabrication. In addition to such high versatility for DLW, nonlinear light-based controls of bottom-up assembly will provide new advances for nanoscience and future technology.

## Conclusions

We report the discovery that hierarchical structures consisting of core and clad layers were formed by femtosecond laser irradiation to AgNO_3_ solution with nanoparticles. TEM observation revealed that the clad layers in the hierarchical cross-sectional structures were filled with nanoparticles. This DLW process was applicable to nanoparticles with different size and optical properties, allowing us to form micropatterns of various materials by laser translation. In particular, we could confine even nanoparticles of SiO_2_, a typical non-photosensitive material, into the clad layers. Such versatile DLW, which overcomes conventional limitations on material choice, will broaden the range of laser materials processing in various scientific and industrial fields.

## Methods

### Femtosecond laser irradiation

We used femtosecond fiber laser (HP-780, Menlosystems. Ltd.), which delivers pulses of 780-nm-wavelength, 127-fs-pulse duration with repetition rate of 100 MHz. Maximum laser power was 80 mW. Linearly polarised laser pulses were focused in the solution through substrates using an objective lens. The numerical aperture of the lens was 0.50 (N20X-PF, Nikon) or 0.60 (UOI-PLACH40xLP, Wraymer, Inc.). Substrates of cover glasses or CaF_2_ plates, after cleaning by acetone, were placed on a Teflon holder filled with the solution. The laser focus was adjusted to the substrate surface in contact with the solution. Micropatterns were written by moving the laser focus using a computer-controlled three-axis stage system. The laser writing process was observed in real time with a CMOS camera system.

### Preparation of solution

Three types of solutions were prepared by adding nanoparticles into a AgNO_3_ solution (Wako Co., Japan) diluted with C_2_H_5_OH (99.5%, Nacalai Tesque, Japan) and deionized H_2_O. Brookite TiO_2_ (NTB-10, Showa-Denko. Co., Japan), SiO_2_ (Sigma-Aldrich Co.) and Fe_2_O_3_ nanoparticles (Sigma-Aldrich Co.) were used. Average diameters of TiO_2_, SiO_2_ and Fe_2_O_3_ nanoparticles were, respectively, 20 nm, 22 nm, 110 nm. Concentration of AgNO_3_, SiO_2_, TiO_2_ and Fe_2_O_3_ were 0.2 M, 2.5 wt%, 1.5 wt% and 1.9 wt%, respectively.

### Material characterization

Surfaces of laser-written microstructures were observed by scanning electron microscope (JSM-5510, JEOL Co., Japan). The elemental analysis was carried out using energy-dispersive X-ray spectroscopy (JSM-7600FA, JEOL Co., Japan). For TEM cross-sectional observation (JEM-2100, JEOL Co., Japan), thin sections of approximately 200 nm thickness were prepared from a line micropattern using focused ion beam (JEM-9320, JEOL Co., Japan). Thin film of Pt-Pd for conductivity and that of C for protection were deposited before FIB etching.

## Supplementary information

direct laser writing

## Data Availability

The datasets during and/or analysed during the current study available from the corresponding author on reasonable request.
